# Effects of subjective and objective task difficulties for feedback- related brain potentials in social situations: An electroencephalogram study

**DOI:** 10.1371/journal.pone.0277663

**Published:** 2022-12-01

**Authors:** Yusuke Yokota, Yasushi Naruse

**Affiliations:** Center for Information and Neural Networks (CiNet), National Institute of Information and Communications Technology, and Osaka University, Kobe, Japan; University of Amsterdam, NETHERLANDS

## Abstract

In this study, the relationship between two types of feedback task difficulties and feedback-related brain potentials, such as feedback-related negativity (FRN), reward positivity (RewP), and P300, was investigated in social situations where participants performed a task simultaneously by a pair. The electroencephalogram activity was measured while participants answered four-choice questions with their partners. Participants were informed about the general accuracy rate of the question (objective task difficulty) before responding to the questionnaire. The feedback outcome was definitely correct when the participants had the knowledge to answer the questions correctly. Therefore, the subjective task difficulty depended on the knowledge of the participant and differed from the objective task difficulty. In the task, the participants selected the choice they deemed correct. Before checking the answers, participants responded to the preceding question’s subjective task difficulty. As one of the social factors, the task consisted of two types of conditions: one, in which one’s response affected partner’s reward, and another, in which it did not. The second social factor was the order of feedback outcomes; in our experiment, these outcomes were presented sequentially to pairs of participants. The effects of subjective and objective task difficulties and social factors on feedback-related brain potentials were comprehensively analyzed. The study showed that subjective task difficulty sensitively modulated the amplitude of gain-related P300, suggesting that it is sensitive to modulation in the allocation of attentional resources to own feedback outcome. The objective task difficulty sensitively modulated the amplitude of RewP after receiving the partner’s incorrect feedback outcome. RewP was more sensitive to positive affective valence, such as feelings of superiority over the partner, than to task-dependent rewards received by the participants themselves. In contrast, FRN was more negative in the joint condition than in the individual condition, suggesting sensitivity to social responsibility felt by participants toward their partners.

## Introduction

We make various decisions to obtain desired outcomes in daily life. In most cases, if one’s action results in a good outcome, it is considered a reward; if it results in a bad outcome, it is considered an error. Sometimes the final judgment is determined not by a decision made by a single person but by decisions made by multiple people. In modern society, social factors are often involved in decision making, and involvement of one’s own and others’ intentions makes the evaluation of rewards and errors more complex. If the task is a simple discrimination task, people can judge at the moment whether the result of their action is good or not. Conversely, when the task involves uncertainty and the outcome is controlled by probability, we cannot detect at the moment whether the outcome is good but can detect results through feedback from the action. One of the event-related potentials (ERP) evoked when feedback is presented is feedback-related negativity (FRN), a fronto-central ERP component strongly observed between 200 and 350 ms after feedback onset [1–3]. FRN has often been observed in monetary reward tasks, and many studies have revealed how FRN is modulated with experimental features such as outcome valence (positive vs. negative), magnitude (large vs. small), and probability (high vs. low). Traditionally, a stronger FRN is evoked with negative than with positive feedback [1–4]. Several studies have found that unexpected negative feedback evokes greater FRN than expected feedback [5–8]. However, the relationship between the outcome magnitude and FRN modulation is controversial, and several studies have argued that FRN is not sensitive to magnitude [3, 4, 9], whereas others have reported that FRN modulates both outcome valence and magnitude [10–13]. Moreover, FRN is sensitive to social factors. For instance, an FRN response is also evoked when another’s error is observed [14–23]. Although observing another’s error evokes an FRN response in cooperative contexts [24–27], observation of others’ success evokes an FRN response in competitive contexts [18, 20, 22, 27, 28]. FRN response is also sensitive to variance in responsibility [29–32], and it changes as per the degree of responsibility when own choices differ from those of others and their choice results in a negative outcome for the group [33–38]. In addition, the FRN response is modulated not only by conflicts with others but also by other components such as processes of emotional and motivational affect [32]. FRN is likely to reflect processing associated with motivational/affective impact rather than the detection of prediction error and cognitive processing of evaluation performance [2, 39].

FRN has been treated differently in previous studies. Some studies have discussed FRN modulation from the differential waveforms between positive and negative feedback. Another study discussed FRN from the viewpoint of negative potential evoked by negative feedback. From here on, we treated negative potential evoked by negative feedback as an FRN component and defined the negative potential generated by the differential waveform between negative deflection after negative feedback and positive deflection after positive feedback as differential feedback-related negativity (dFRN). Several studies found that dFRN’s amplitude is directly attributed not to the negative potential caused by negative feedback but rather to the positive potential caused by positive feedback [13, 40–43]. One report hypothesizes that unexpected feedback evokes negative potential as N200 regardless of whether the outcome valence is positive or negative [44], and expected positive feedback evokes a positive potential referred to as reward positivity (RewP) [42, 44, 45]. As a result, positive deflection attenuates N200. Traditionally, stronger RewP is caused by improbable positive (reward) feedback rather than probable positive feedback, and these modulations were more sensitive than instances with negative feedback [40, 45–47]. Furthermore, RewP has been reported to be associated with reward magnitude [48, 49]. Negative potential evoked by negative feedback and RewP is considered to be caused by different neural mechanisms. Therefore, the modulation of the FRN evoked by negative feedback and the RewP evoked by positive feedback should be discussed as independent ERP components.

Another ERP component that encodes the neural response associated with the feedback outcome is P300, which is observed between 300 and 600 ms after feedback onset. P300 has been reported to be associated with various cognitive processes [4, 50, 51]. The P300 amplitude observed for unexpected feedback outcome is higher than that observed for expected feedback outcome [3, 11, 52–54]. Several studies reported that P300 response is modulated by feedback magnitude but not by feedback valence [10, 55–57]. Furthermore, several studies have reported that the P300 response for gain-related feedback is larger than that for loss-related feedback [40, 58, 59], several other studies have reported the opposite trend [3, 11–13, 33, 53, 57, 60–62]. P300 reflects not only attentional allocation to the stimulus [63–65] but also modulation of social contexts such as interpersonal relationships and the level of personal responsibility for the feedback outcome [24–26, 32–34, 38, 66–68].

This study focused on novel perspectives of neural processing associated with positive and negative feedback processes. Several previous studies have discussed feedback-related neural processes in terms of positive/negative feedback by employing a gambling task of choosing one of multiple alternatives [3, 4, 9, 34, 35, 66, 69]. In most previous studies, feedback probabilities were determined by experimental conditions. In other words, participants expected upcoming outcomes according to probabilities determined by a computer program. In this case, participants were not fundamentally responsible for the outcome of their choice because it depended entirely on probability. In contrast, cue-learning [70–73] and AIOWA gambling tasks [74–78], whereby participants are able to implicitly predict the reward probabilities of multiple alternatives, have also been used to investigate feedback neural processes. In these tasks, participants are to some degree responsible for the outcome because they can choose the alternative with the highest reward probability depending on their own learning. Nevertheless, the feedback results are still governed by the probabilities, and participants cannot clearly know the reward probability and their own degree of responsibility for the task. In the present study, we used a task in which their choice, according to their amount of knowledge, resulted in good or bad outcomes. In particular, we employed a quiz format with a question text and four corresponding choices. The feedback outcome of the choice was determined by participants’ amount of knowledge and luck. Therefore, feedback involved not only uncertainty as a probability but also certainty because participants could choose correctly if they had enough knowledge. Thus, they took some responsibility for their choice. In the task, we presented the participants with the questions’ general accuracy rate (GAR) along with corresponding questions to adjust participants’ amount of responsibility. The GAR was the percentage of the total number of people who answered the question correctly. In contrast, if participants had the knowledge to answer the question, their self-confidence rate (SCR) for feedback outcomes was extremely high and vice versa. Most previous studies that focused on FRN, RewP, and P300 experimented with a gambling task, and their participants predicted the feedback outcome according to a presented reward (or penalty) probability. In contrast, the task of the present study, where participants’ amount of knowledge affected the certainty for feedback outcomes, the GAR was an objective task difficulty, and the participants’ subjective confidence rate to feedback outcomes corresponded to a subjective task difficulty. To the best of our knowledge, few studies have investigated the relationship between feedback-related ERPs and task difficulty. Several studies have reported that no effect of the task difficulty was shown on the FRN [79–81]. Another study has reported the opposite evidence [82]. In contrast, P300 was found to be sensitive to task difficulty [80, 81]. Research on feedback-related ERPs and task difficulty is still insufficient. Moreover, there is a lack of research investigating how feedback-related ERPs are modulated in tasks where subjective and objective task difficulties are separated.

Moreover, we aimed to elucidate the affection of social factors to feedback-related potentials. Here, social factors refer to the actual or potential impact that the feedback outcome of self has on others or that others’ feedback outcome has on the self. The present task was performed simultaneously by a pair, and they were informed of each other’s feedback outcomes to induce social factors. The first source of social factors was the experimental conditions. We used a joint condition, wherein two participants were rewarded if both choices were correct, and an individual condition, wherein participants were rewarded if their own choice was correct. Therefore, one’s feedback outcome in the joint condition affected not only oneself but also others’ gain or disadvantage. The second source of social factors was the order of feedback outcomes, which were presented sequentially in our experiment. When one’s feedback results were presented after presenting the partner’s feedback outcomes, the effect of the responsibility one feels for one’s own feedback outcome may vary depending on the correctness or incorrectness of the prior partner’s outcome. For instance, if the feedback outcomes of a prior partner in a joint condition were correct, the sense of responsibility felt for one’s subsequent feedback outcome should be large. In this case, we hypothesized that a larger FRN is observed in the joint condition than in the individual condition if own answer was incorrect. In contrast, if a prior partner’s feedback outcomes were incorrect, a sense of superiority might arise if one’s own subsequent feedback outcomes were correct. Thus, in this case, we hypothesized that a larger RewP would be evoked. Because previous studies have reported that P300 is sensitive to task difficulty [80, 81], we hypothesized that P300 amplitude is probably modulated by either subjective or objective task difficulty. To the best of our knowledge, no studies on the modulation of feedback-related ERPs in the presence of social factors in addition to the separation of feedback task difficulty into subjective and objective task difficulties have been reported.

The present study investigated the modulation of ERPs’ amplitude (FRN, RewP, and P300) with experimental task difficulties (subjective/objective outcome task difficulties) and social factors at a single-trial level. The novelty of this study is that we comprehensively analyzed effects of subjective and objective task difficulties that occurred during tasks that involve responsibility for the outcome of one’s actions in the social situation.

## Material and methods

### Creating general-knowledge quiz questions

**Fig 1:**
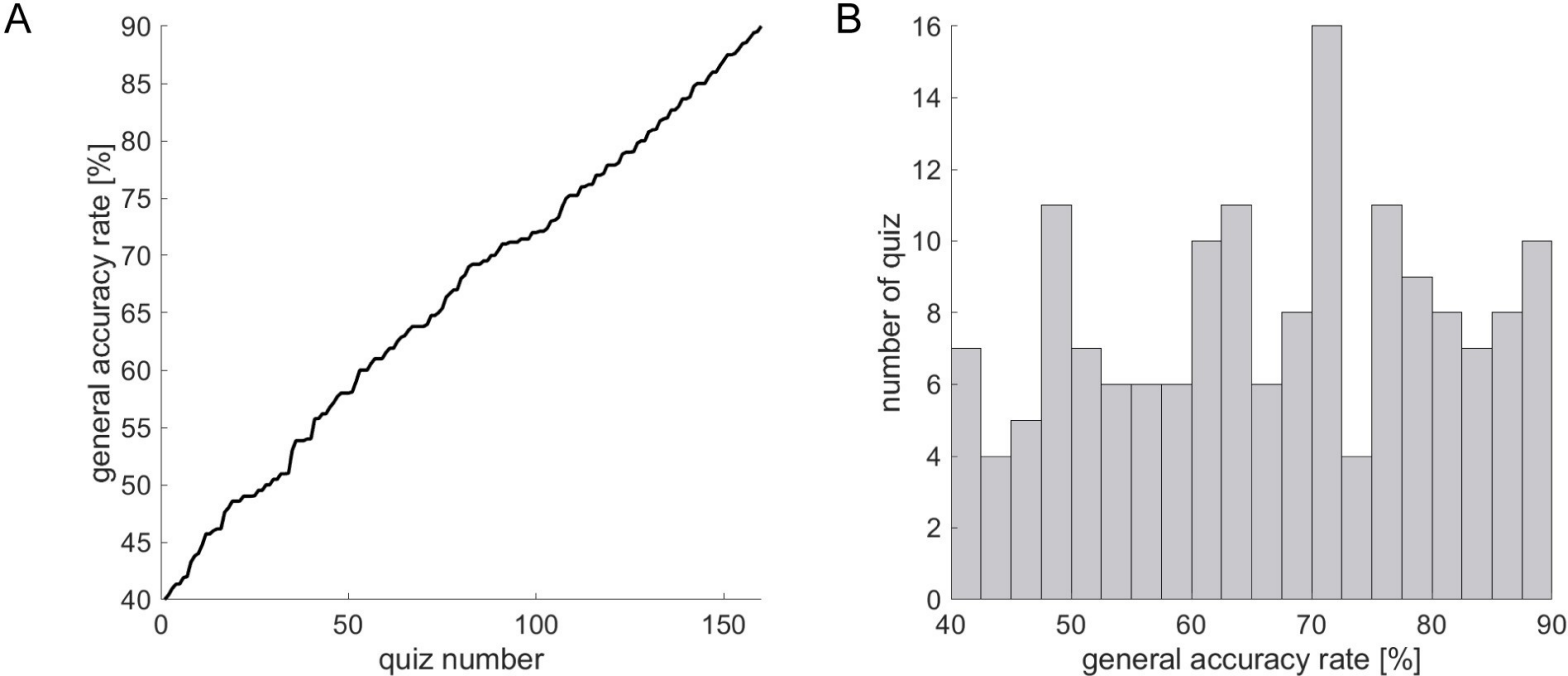
(A) General accuracy rate of 160 questions. (B) Histogram of general accuracy rate of 160 questions.

Consisting of generally known facts and knowledge from trivia in literature, language, history, geography, and science, our quiz was administered to participants as four-choice questions, with participants choosing the answer they deemed correct. We prepared 300 unique questions for the experiment. To calculate the GAR for each question, 353 men and women in the age range of 20–39 answered the questions in advance. Because the survey was conducted via a website, we could not set a time limit to answer each question. However, we asked participants to answer the questions within an approximate response time of 10 s per question. As data cleaning after the survey, we excluded the data of answerers whose total time to answer all questions as extremely short (3 s per question) or long (15 s per question) from the subsequent analysis. Consequently, the data from 41 answerers were excluded from subsequent analyses. We then defined the GAR as the number of correct answers divided by the number of total answers, and the GAR was calculated for each question. Finally, this study used 160 questions with the GAR ranging from 40% to 90% for all 300 questions. Fig 1A shows the GAR distribution for the 160 selected questions, and Fig 1B shows the histogram. The mean of the GAR was 66.5% (SD = 13.9%).

### Participants

Sixty-four individuals (32 men, 32 women; age range: 20–39 years) participated in this study. All participants had normal hearing and normal or corrected-to-normal vision and had earned a junior college, college, or university degree or were enrolled in a degree program. Participants provided written informed consent after the procedural details had been explained and before the experiment. All experimental procedures were approved by the Ethics Committee for Human and Animal Research of National Institute of Information and Communications Technology. All experiments were performed in accordance with the ethical standards described in the Declaration of Helsinki. The data of two participants were removed from the subsequent analysis because of technical problems during the measurements.

### Experimental procedure

**Fig 2:**
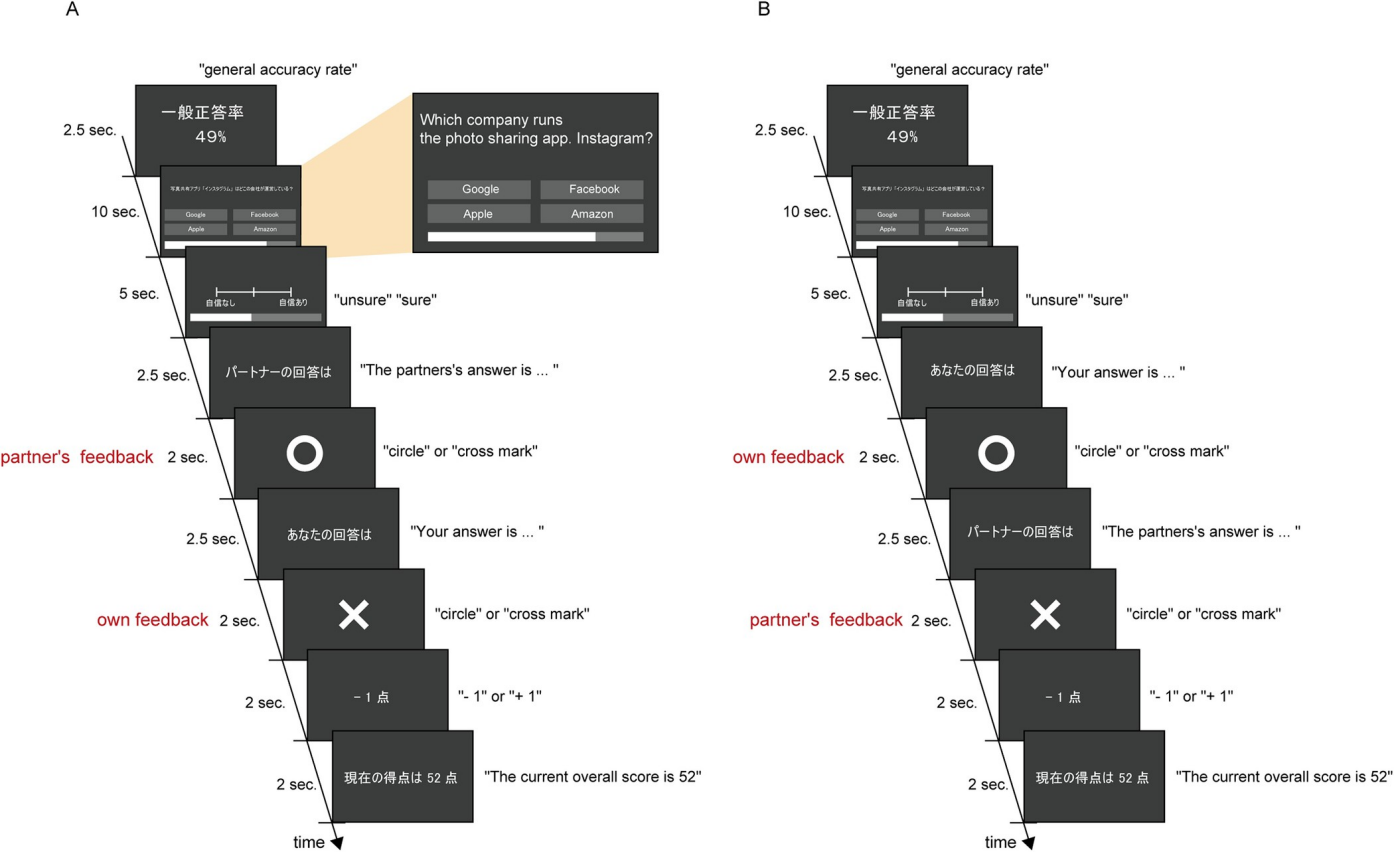
Experimental procedure. The participants paired up and performed a four-choice question task simultaneously. Participants were presented with the question text, the general accuracy rate of the question, and four corresponding choices. After answering, the participant reported the degree of confidence rate for the question.

Fig 2 schematically illustrates the experimental procedure. In the experiment, two participants joined simultaneously in answering the same question (Fig 2A and 2B). The pairs belonged to the same gender, and their age difference was less than 5 years. First, a GAR for each question was presented for 2.5 s. Then the question text and its four choices were presented for 10 s. Participants were instructed to click the left mouse button on the choice they deemed correct. When the participant clicked on a choice, the text of the corresponding answer choice turned red. Participants were not allowed to change their choices after selecting them. At the bottom of the screen, a bar was presented to indicate the time limit for the choice. If participants did not select any option within the time limit (10 s), the feedback outcome was treated as incorrect, and the trials were excluded from subsequent analysis. Participants then responded to the preceding question’s degree of confidence through a visual analog scale (VAS), defined in this study as the SCR. SCRs were coded as a quantitative value between 0 and 1. SCRs were indicated by clicking the left mouse button through a VAS, and the time limit was 5 s. At the bottom of the screen, a bar was also presented to indicate the time limit for the response. Trials in which participants did not respond to the SCR were excluded from analysis. Next, the judgment (i.e., feedback) of the two participants’ choices was presented sequentially. The order of feedback was the common aspect between the two participants. Therefore, if participant A’s answer was presented first, the stimulus “Your answer is…” was presented on participant A’s screen (Fig 2A), and the stimulus “Your partner’s answer is…” was presented on participant B’s screen (Fig 2B). This advance notice was presented for 2.5 s, after which the feedback outcome was indicated by a circle (generally indicating correct) or a cross mark (indicating incorrect in Japan). Next, the score that was +1 or –1 according to the feedback outcome was shown, and finally, the current overall score was shown. The background of the screen was presented in RGB (60, 60, 60) gray, and visual stimuli of letters and feedback marks were presented in RGB (255, 255, 255) white. We attached experimental scripts (S1 Appendix) into the supporting information.

We used joint and individual conditions as the experimental condition. In the joint condition, both participants’ scores were added only when both answered correctly; when either participant answered incorrectly, both scores were subtracted. In the individual condition, a participant’s score was independent of the partner’s choice, and the score was added or subtracted depending on own choice. Therefore, the feedback outcome of the partners’ choice did not affect own scores. Each participant was given 50 points at the beginning; then in both conditions, one point was added with each correct outcome and subtracted with each incorrect outcome.

The experiment consisted of four blocks. Forty questions were presented in each block; therefore, participants answered 160 questions throughout the experiment. The blocks’ order was counterbalanced by the experimental condition (joint or individual) and the order of feedback outcome between participants and their partners. Experimental conditions and the order of feedback were presented to participants before the start of the experimental block.

### Experimental devices

We measured electroencephalogram (EEG) responses using a portable EEG device (Miyuki Giken, original development based on Polymate Mini AP108 [W52-D50-H20 mm, 80 g], JPN) with active dry electrodes (Unique Medical, unique development, Japan) positioned at FC3, FCz, and FC4 according to the International 10–20 system. To obtain electrooculograms (EOGs), two electrodes were placed on the top and side of participants’ right eyes. All recorded signals were referenced to the right mastoid, and the ground electrode was placed on the left mastoid. EEG and EOG data were sampled at 500 Hz.

The visual-stimulus presentation was controlled in Matlab 2019a (MathWorks, Inc., Natick, MA, USA), using the Psychophysics Toolbox extensions [83, 84], and was presented on liquid-crystal displays (Eizo, ColorEdge CX241, JPN, framerate: 60 Hz). Pairs of participants sat in chairs back to back about 2 m apart and observed the stimuli at approximately 50 cm from the monitors, respectively.

### EEG data analyses

EEG analyses were performed using MATLAB 2019a. A digital finite-impulse response bandpass filter (1–20 Hz, order 1500) was applied to continuous EEG and EOG signals. Subsequently, we used independent component analysis to remove from the EEG data artifacts associated with eye movement. EEG data were divided into 1200-ms epochs (−200 to +1000 ms) based on own feedback onsets after partners’ feedback. Baseline correction was performed using the averaged amplitude from −200 to 0 ms. Trials exceeding ±40 μV on the FC3, FCz, and FC4 channels were excluded from analysis.

### Statistical analyses

The study used a linear mixed-effects model (LME) for statistical analysis. In traditional neuroscience studies for ERPs, EEG data are averaged for each experimental condition and participant; analysis of variance (ANOVA) is often used to test the statistical significance between experimental conditions. This traditional technique is based on the hypothesis that all participants and experimental conditions average the same number of trials. However, because the number of trials in our study depended on each participant’s choice, they differed to a large extent for each participant. In addition, the GAR for each question and that SCR for each participant differed in each trial. Therefore, using the traditional ANOVA for the current experimental data was not a desirable approach. Because LME is robust for an unequal number of observations for each experimental condition and participant, we used an LME model instead of the traditional ANOVA.

The LME model estimates regression weights of fixed and random effects. To identify effects of FRN, RewP, and P300 responses, we defined own feedback outcome (OFB), partners’ feedback outcome (PFB), experimental conditions (ExpCond), GAR, and SCR as fixed effects. We also defined ERP differences among participants as a random intercept (Participants). The OFB, PFB, and ExpCond were categorical variables, whereas GAR and SCR were continuous variables. The categorical variables were coded as 0/1 and the continuous variables were transformed into z-scores. As we described in the Introduction section, FRN is observed between 200 and 300 ms, whereas P300 is observed between 300 and 600 ms after feedback onset. However, the actual time windows used varied considerably between previous studies (for example, see [85]). Hence, we visually detected the time windows for FRN and P300 that were considered to have little overlap with other ERP components from the grand-averaged waveform and subsequently used them for the statistical analyses. The values of FRN and RewP for LME were calculated by mean amplitudes between 200 and 250 ms after the correct and incorrect OFB onset in the averaged data of the FC3, FCz, and FC4 channels. Similarly, the value of loss- and gain-related P300s for LME analysis was calculated by mean amplitude from 300 to 400 ms. To investigate the effect of the relationship between GAR/SCR and social factors, the study analyzed brain activities evoked by OFB onset after PFB. The models for each ERP in the Wilkinson notation are ERPs amplitude ~ OFB + OFB:(PFB * ExpCond * (SCR +GAR)) + (1|Participant).

We used Rstudio version 1.3.1056 for statistical analysis and performed LME analysis using the *lme4* and *lmerTest* package. The type III ANOVA test with the Kenward–Roger method was used to determine the significance of the fixed effects. For post-hoc tests, multiple comparisons were performed based on fitted LMEs using the Kenward–Roger. The Tukey method was used to correct the p-values for multiple comparisons wherever necessary. For the significant interaction of ERP responses, the significance of a GAR coefficient and SCR trends per level of factor and differences in coefficients across levels were tested. All post-hoc tests were performed using *emmeans* packages. We used the *confint* function of the MASS package to calculate confidence intervals of each prediction. We attached a script (S2 Appendix) and data (S3 Appendix) for statistical analyses into the supporting information. The fifth sheet in S3 Appendix contains the segmented ERP results from specific time windows used in the analysis.

## Results

### Behavioral analysis

**Fig 3:**
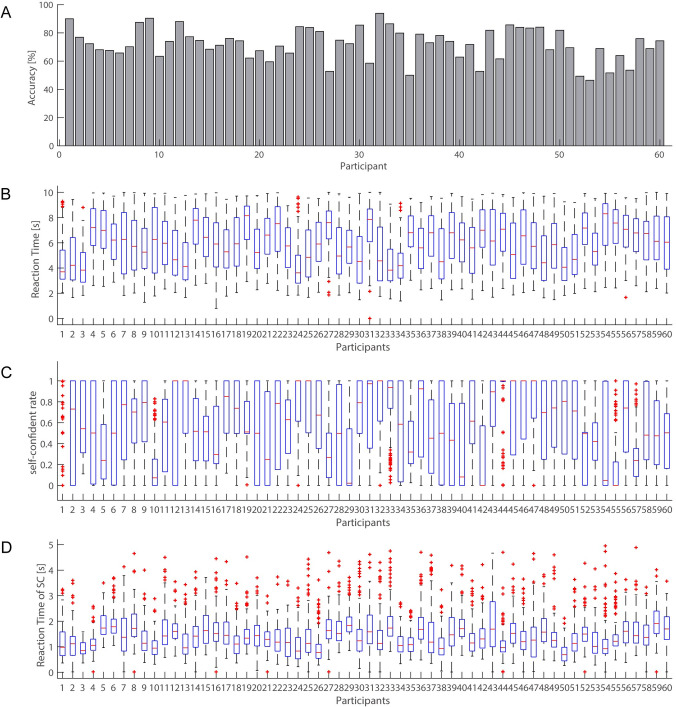
Behavioral result. (A) Averaged accuracy. (B) Averaged reaction time. (C) Averaged SCR. (D) Averaged reaction time for SCR response.

The averaged accuracy and reaction time for questions across participants were 72.2% (SD = 11.3) and 5.90 s (SD = 0.884), respectively. The averaged SCR and their reaction time for SCR response across participants were 0.557 (SD = 0.164) and 1.45 s (SD = 0.297), respectively. Fig 3 shows each participant’s behavioral results.

### EEG analysis

**Fig 4:**
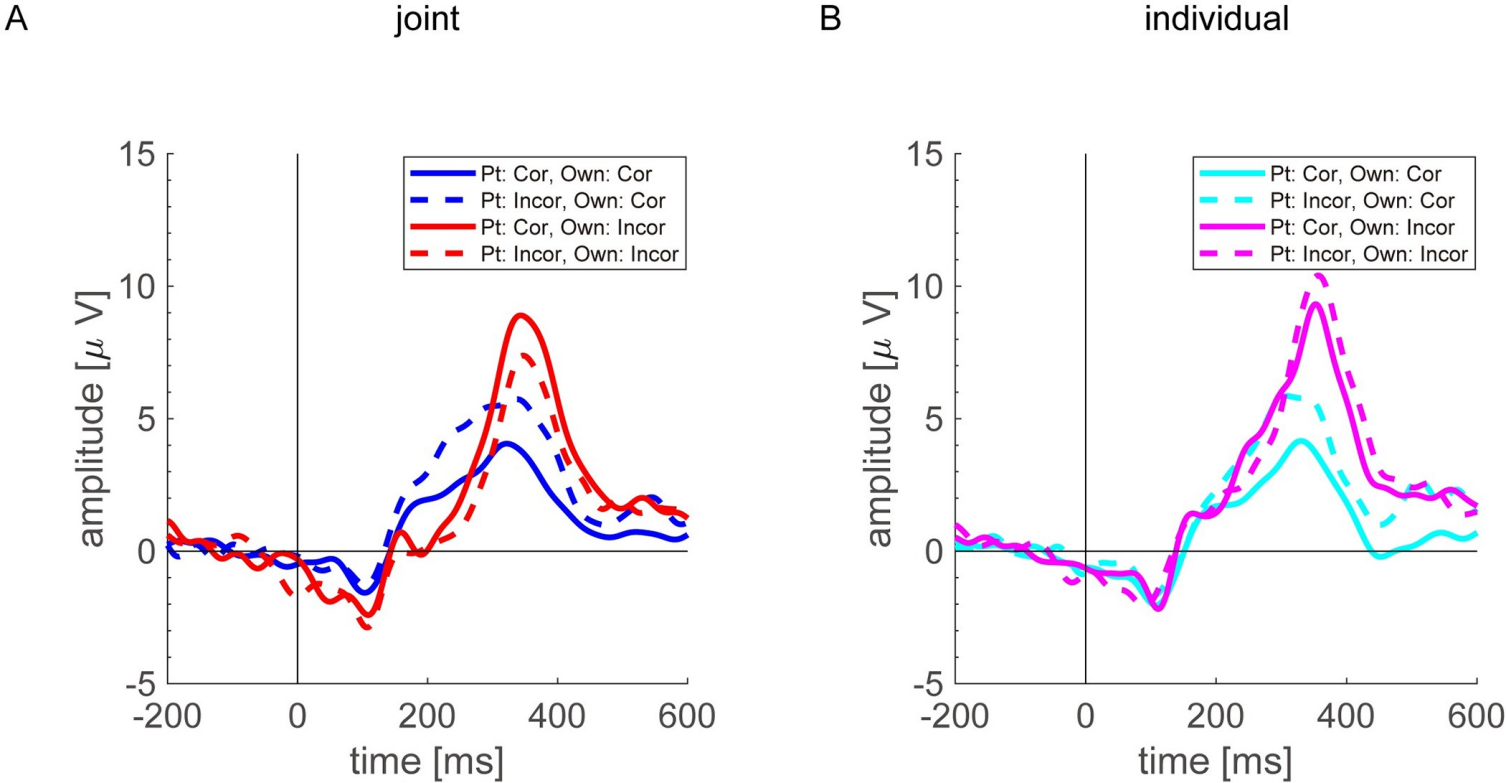
Grand-averaged event-related potentials of the averaged data of the FC3, FCz, and FC4 channels in (A) joint and (B) individual conditions.

Fig 4 shows grand-averaged feedback ERPs of the averaged data of the FC3, FCz, and FC4 channels. Fig 4A represents waveforms of joint condition, and Fig 4B represents waveforms of individual condition. In case of incorrect own feedback, we confirmed negative deflection after event onset (+200 to +250 ms). We treated negative deflection in the incorrect feedback outcome as the FRN. In the correct feedback, we confirmed positive deflection at the same latency as the FRN after event onset compared with incorrect feedback. We treated positive deflection in the correct feedback outcome as the RewP. Moreover, we confirmed positive deflections after event onset (+300 to +400 ms) in correct and incorrect feedback, respectively. We treated positive deflections as gain-related P300 and loss-related P300, respectively.

### Statistical analysis

**Fig 5:**
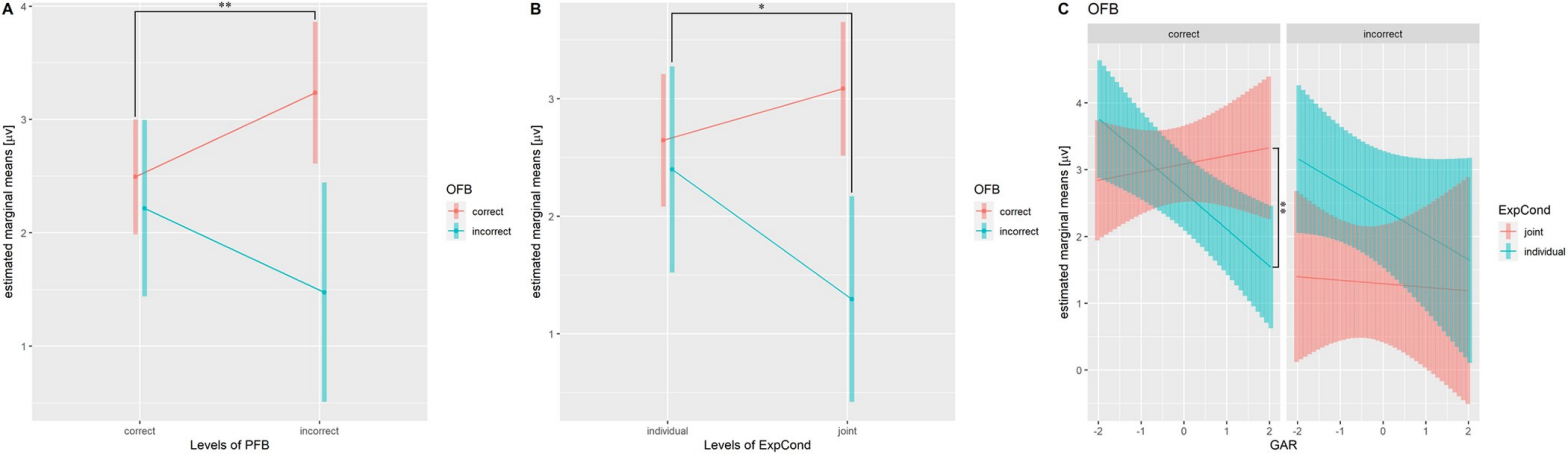
Estimated marginal means of FRN/RewP. (A) Interaction of OFB and PFB. (B) Interaction of OFB and ExpCond. (C) Interaction of OFB, ExpCond, and GAR. *p < 0.05, **p< 0.01.

Table 1 shows the total number of trials and the average number of trials among participant for each condition applied to the LME fitting. Tables 2 and 3 show summaries of LME fitting (estimated coefficients, f-test) for FRN/RewP. Table 4 and Fig 5 show results of post-hoc analysis and significant interaction terms. ANOVA revealed significant interactions [OFB:PFB, F (2) = 4.87, p = 0.008; OFB: ExpCond, F (2) = 3.47, p = 0.031; and OFB: ExpCond: GAR, F (2) = 3.41, p = 0.033].

**Table 1:** Total number of trials and the average number of trials among participant for each condition.

Condition (own feedback: partner’s feedback)	Total number	*Average number*	*SD*
correct: correct	2358	39.3	12.00
correct: incorrect	732	12.2	7.18
incorrect: correct	660	11	6.22
incorrect: incorrect	490	8.17	4.08

**Table 2:** Estimated coefficients of fixed effects in LME for FRN/RewP.

Fixed effects	Estimates	*SE*	*CI*
Intercept	2.44	0.293	[1.87, 3.02]
OFB (incorrect)	0.0576	0.480	[−0.880, 0.998]
OFB (correct): PFB (incorrect)	0.408	0.369	[−0.312, 1.13]
OFB (incorrect): PFB (incorrect)	−0.198	0.771	[−1.71, 1.31]
OFB (correct): ExpCond (joint)	0.0998	0.265	[−0.418, 0.618]
OFB (incorrect): ExpCond (joint)	−0.561	0.641	[−1.81, 0.692]
OFB (correct): SCR	−0.578	0.191	[−0.952, −0.203]
OFB (incorrect): SCR	0.0236	0.422	[−0.801, 0.851]
OFB (correct): GAR	−0.484	0.183	[−0.840, −0.127]
OFB (incorrect): GAR	−0.546	0.309	[−1.15, 0.0577]
OFB (correct): PFB (incorrect): ExpCond (joint)	0.671	0.520	[−0.347, 1.69]
OFB (incorrect): PFB (incorrect): ExpCond (joint)	−1.09	1.09	[−3.21, 1.03]
OFB (correct): PFB (incorrect): SCR	0.757	0.363	[0.0476, 1.47]
OFB (incorrect): PFB (incorrect): SCR	0.562	0.667	[−0.743, 1.87]
OFB (correct): PFB (incorrect): GAR	−0.138	0.355	[−0.833, 0.556]
OFB (incorrect): PFB (incorrect): GAR	0.335	0.513	[−0.669, 1.34]
OFB (correct): ExpCond (joint): SCR	0.381	0.265	[−0.137, 0.899]
OFB (incorrect): ExpCond (joint): SCR	0.447	0.601	[−0.729, 1.62]
OFB (correct): ExpCond (joint): GAR	0.435	0.263	[−0.0785, 0.950]
OFB (incorrect): ExpCond (joint): GAR	0.207	0.487	[−0.745, 1.16]
OFB (correct): PFB (incorrect): ExpCond (joint): SCR	−0.983	0.512	[−1.98, 0.0178]
OFB (incorrect): PFB (incorrect): ExpCond (joint): SCR	−0.768	0.962	[−2.65, 1.11]
OFB (correct): PFB (incorrect): ExpCond (joint): GAR	0.478	0.542	[−0.583, 1.54]
OFB (incorrect): PFB (incorrect): ExpCond (joint): GAR	0.238	0.809	[−1.34, 1.82]

**Table 3:** Summaries of the type III analysis of variance for FRN/RewP.

	*F*.*value*	*df*	*p val*
OFB	0.01	1	0.904
OFB: PFB	4.87	2	0.008*
OFB: ExpCond	3.47	2	0.031*
OFB: SCR	2.85	2	0.058
OFB: GAR	1.81	2	0.164
OFB: PFB: ExpCond	1.33	2	0.264
OFB: PFB: SCR	0.60	2	0.549
OFB: PFB: GAR	0.70	2	0.497
OFB: ExpCond: SCR	0.10	2	0.904
OFB: ExpCond: GAR	3.41	2	0.033*
OFB: PFB: ExpCond: SCR	2.16	2	0.125
OFB: PFB: ExpCond: GAR	0.43	2	0.650

**Table 4:** Summaries of post-hoc test for FRN/RewP.

*OFB*	*contrast (PFB)*	*estimate*	*SE*	*t*.*ratio*	*p*.*val*
correct	correct–incorrect	−0.74	0.27	−2.78	0.005*
incorrect	correct–incorrect	0.74	0.55	1.36	0.175
***OFB***	***contrast (ExpCond)***	***estimate***	***SE***	***t*.*ratio***	***p*.*val***
correct	individual–joint	−0.44	0.26	−1.69	0.092
incorrect	individual–joint	1.10	0.54	2.04	0.042*
***ExpCond***	***OFB***	***GAR*.*trend***	***SE***	***t*.*ratio***	***p*.*val***
individual	correct	−0.55	0.18	−3.09	0.002**
joint	correct	0.12	0.21	0.59	0.554
individual	incorrect	−0.38	0.26	−1.47	0.142
joint	incorrect	−0.05	0.31	−0.17	0.865
***OFB***	***contrast (ExpCond)***	***diff*.*GAR***	***SE***	***t*.*ratio***	***p*.*val***
correct	individual–joint	−0.67	0.27	−2.49	0.013*
incorrect	individual–joint	0.33	0.40	−0.81	0.421

Multiple comparisons revealed that the amplitude of incorrect OFB in the joint condition were significantly lower than those in the individual condition [t = 2.04, p = 0.042], indicating that FRN amplitude was more negative in joint conditions than in individual conditions. In contrast, multiple comparisons revealed that the correct OFB amplitude with incorrect PFB were significantly higher than those with correct PFB [t = −2.78, p = 0.005]. The result indicated that RewP amplitude was more positive with incorrect PFB than those with correct PFB. Besides, RewP amplitudes in the correct OFB and the GAR trend showed a significant negative correlation in the individual condition [t = −3.09, p = 0.002], and this trend was significantly more negative than that in the joint condition [t = −2.49, p = 0.013].

**Fig 6:**
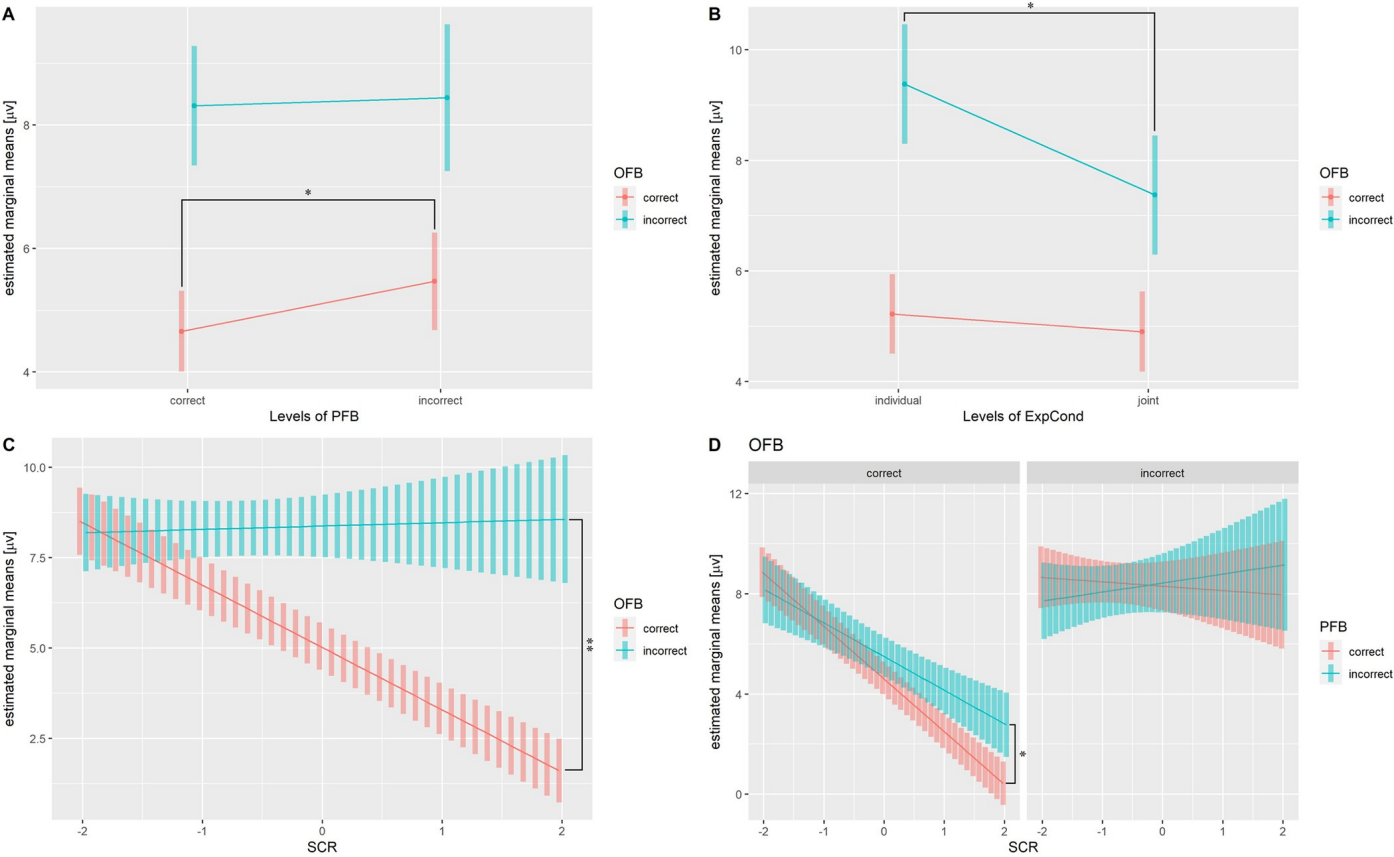
Estimated marginal means of gain- and loss-related P300s. (A) Interaction of OFB and PFB. (B) Interaction of OFB and ExpCond. (C) Interaction of OFB and SCR. (D) Interaction of OFB, OFB, and SCR. *p < 0.05, **p< 0.01.

Tables 5 and 6 show summaries of LME fitting (estimated coefficients, f-test) for loss- and gain-related P300s. Table 7 and Fig 6, respectively, show the results of post-hoc analysis and significant interaction terms. ANOVA revealed the effect of PFB and significant interactions [OFB: PFB, F (2) = 3.20, p = 0.041; OFB: ExpCond, F (2) = 5.29, p = 0.005; OFB: SCR, F (1) = 57.82, p < 0.001; and OFB: PFB: SCR, F (2) = 3.45, p = 0.032].

**Table 5:** Estimated coefficients of fixed effects in LME for gain- and loss-related P300.

Fixed effects	Estimates	*SE*	*CI*
Intercept	4.75	0.372	[4.01, 5.48]
OFB (incorrect)	3.95	0.577	[2.82, 5.078]
OFB (correct): PFB (incorrect)	0.939	0.443	[0.0732, 1.80]
OFB (incorrect): PFB (incorrect)	1.37	0.927	[−0.444, 3.18]
OFB (correct): ExpCond (joint)	−0.192	0.318	[−0.815, 0.429]
OFB (incorrect): ExpCond (joint)	−0.773	0.770	[−2.28, 0.732]
OFB (correct): SCR	−2.26	0.230	[−2.70, −1.80]
OFB (incorrect): SCR	0.274	0.507	[−0.717, 1.27]
OFB (correct): GAR	−0.711	0.219	[−1.14, −0.283]
OFB (incorrect): GAR	0.0511	0.371	[−0.675, 0.776]
OFB (correct): PFB (incorrect): ExpCond (joint)	−0.259	0.625	[−1.48, 0.963]
OFB (incorrect): PFB (incorrect): ExpCond (joint)	−2.48	1.30	[−5.02, 0.0721]
OFB (correct): PFB (incorrect): SCR	1.14	0.436	[0.285, 1.99]
OFB (incorrect): PFB (incorrect): SCR	0.892	0.801	[−0.675, 2.46]
OFB (correct): PFB (incorrect): GAR	0.451	0.427	[−0.384, 1.29]
OFB (incorrect): PFB (incorrect): GAR	−0.729	0.617	[−1.93, 0.477]
OFB (correct): ExpCond (joint): SCR	0.300	0.318	[−0.323, 0.922]
OFB (incorrect): ExpCond (joint): SCR	−0.896	0.722	[−2.31, 0.515]
OFB (correct): ExpCond (joint): GAR	0.257	0.316	[−0.361, 0.875]
OFB (incorrect): ExpCond (joint): GAR	−0.693	0.585	[−1.84, 0.451]
OFB (correct): PFB (incorrect): ExpCond (joint): SCR	−0.752	0.615	[−1.95, 0.449]
OFB (incorrect): PFB (incorrect): ExpCond (joint): SCR	−0.716	1.16	[−2.98, 1.54]
OFB (correct): PFB (incorrect): ExpCond (joint): GAR	0.326	0.652	[−0.948, 1.60]
OFB (incorrect): PFB (incorrect): ExpCond (joint): GAR	2.02	0.971	[0.120, 3.92]

**Table 6:** Summaries of the type III analysis of variance for gain- and loss-related P300.

	*F*.*value*	*df*	*p val*
OFB	46.90	1	0.000**
OFB: PFB	3.20	2	0.041*
OFB: ExpCond	5.29	2	0.005*
OFB: SCR	57.82	2	0.000**
OFB: GAR	1.61	2	0.199
OFB: PFB: ExpCond	1.89	2	0.151
OFB: PFB: SCR	3.45	2	0.032*
OFB: PFB: GAR	1.93	2	0.145
OFB: ExpCond: SCR	2.38	2	0.093
OFB: ExpCond: GAR	1.04	2	0.354
OFB: PFB: ExpCond: SCR	0.94	2	0.391
OFB: PFB: ExpCond: GAR	2.28	2	0.102

**Table 7:** Summaries of post-hoc test for gain- and loss-related P300.

*OFB*	*contrast (PFB)*	*estimate*	*SE*	*t*.*ratio*	*p*.*val*
correct	correct–incorrect	−0.81	0.32	−2.52	0.012*
incorrect	correct–incorrect	−0.13	0.66	−0.20	0.845
***OFB***	***contrast (ExpCond)***	***estimate***	***SE***	***t*.*ratio***	***p*.*val***
correct	individual−joint	0.32	0.31	1.02	0.307
incorrect	individual−joint	2.00	0.65	3.08	0.002**
***OFB***		***SCR*.*trend***	***SE***	***t*.*ratio***	***p*.*val***
correct		−1.73	0.16	−10.72	0.001**
incorrect		0.09	0.30	0.31	0.757
***PFB***	***OFB***	***SCR*.*trend***	***SE***	***t*.*ratio***	***p*.*val***
correct	correct	−2.11	0.17	−12.49	0.001**
incorrect	correct	−1.34	0.27	−5.04	0.001**
correct	incorrect	−0.17	0.37	−0.47	0.640
incorrect	incorrect	0.36	0.46	0.79	0.431
***OFB***	***contrast (PFB)***	***diff*.*SCR***	***SE***	***t*.*ratio***	***p*.*val***
correct	correct–incorrect	−0.76	0.31	−2.46	0.014*
incorrect	correct–incorrect	−0.53	0.58	−0.92	0.357

Multiple comparisons revealed that the amplitude of incorrect OFB in the individual condition was significantly higher than that in the joint condition [t = 3.08, p = 0.002], indicating that loss-related P300 amplitude was more positive in individual conditions than in joint conditions. In contrast, multiple comparisons revealed that correct OFB amplitudes with correct PFB were significantly higher than those of incorrect PFB [t = −2.52, p = 0.012]. The result indicated that gain-related P300 amplitude was more positive with incorrect PFB than with correct PFB. Besides, the amplitude of the correct OFB and the SCR trend showed a significant negative correlation [t = −10.72, p = 0.001], and the trend with correct PFB was significantly more negative than that with incorrect PFB [t = −2.46, p = 0.014].

## Discussion

The present study investigated the effect of social factors generated by paired participants simultaneously answering questions and sharing each other’s outcomes on ERP components related to their feedback. We elucidated how factors such as experimental task difficulties (SCR and GAR) and social factors (PFB and ExpCond) modulated FRN, RewP, and loss- and gain-related P300.

### FRN/RewP

First, statistical analysis revealed significant interactions in ERP for FRN/RewP between OFB and ExpCond. For the post-hoc test, the amplitude of the ERP that was evoked in the incorrect OFB was more negative in the joint condition than in the individual condition. Therefore, FRN that was a negative deflection evoked by incorrect OFB was significantly different among the ExpCond. This finding can be explained by the personal responsibility toward one’s partner. Pu and Yu [38] reported that when a participant’s choice led to negative feedback outcomes, the amplitude of FRN changed according to the magnitude of personal responsibility. Similarly, Kimura et al. [37] reported that when participants felt stronger responsibility, for instance, when they were empowered to make a decision on voting, a stronger FRN was observed if their own voting resulted in negative feedback. Kimura and Katayama [34] reported that FRN amplitude for negative feedback outcome was negatively larger when one’s own choice led to monetary loss for other participants. These reports indicated that FRN amplitude was sensitively modulated by the magnitude of personal responsibility. In this study, more negative FRN amplitudes in the joint condition than in the individual condition, indicating that these results were comparable to those of previous studies [34, 37] in which an error in situations when participants were responsible for their choices enhanced the negative potentials of FRN.

Next, statistical analysis revealed significant interactions in ERP for FRN/RewP between OFB and PFB. For the post-hoc test, the amplitude of the ERP that was evoked in the correct OFB was more positive in the incorrect PFB than in the correct PFB. Therefore, RewP that was a positive deflection evoked by correct OFB was significantly different among the PFB. Larger RewP was observed in one’s correct OFB after an incorrect PFB than in a correct PFB. Several previous studies have supported our result. Li et al. [33] reported that a stronger negative dFRN is evoked in a situation of high self-responsibility. However, we consider from the figure in their report that modulation was caused by positive amplitude in positive feedback. Yu and Sun [32] reported that a stronger negative dFRN between FRN and RewP was found in the experimental condition in which participants chose different options from those chosen by others than in the experimental condition in which they made the same choices as others. However, we also believe from the figure in their report that this amplitude modulation was caused by changes in RewP rather than in FRN. Moreover, Kimura and Katayama [35] used a majority voting task with three participants and reported that gain-related ERPs were larger when participants chose different options from those chosen by others, whereas loss-related ERPs showed no significant difference. Similarly, Kimura et al. [37] reported that a larger RewP amplitude was observed in the positive-feedback outcome in a majority of trials than in minority trials. In this study, the facts that FRN and RewP modulations were observed for both incorrect and correct OFB, respectively, suggest that participants elicited greater emotional valence from both negative- and positive-feedback outcomes. In particular, a larger RewP was observed in the correct OFB after the incorrect PFB suggests that RewP amplitude was sensitive to positive emotions such as superiority to the partner.

Next, we mentioned the relationship between subjective/objective task difficulties and modulation of FRN/RewP amplitudes. Statistical analysis revealed significant interactions between ExpCond and GAR in RewP. For the post-hoc test, the correct OFB in the joint condition did not correlate significantly with the GAR, whereas the correct OFB in the individual condition did correlate significantly negatively with the GAR. Besides, we observed a significant difference between slopes of the GAR in joint and individual conditions in the correct OFBs. In the individual condition, RewP amplitude was similar to that in the joint condition for questions that were more difficult in which GARs were low, but amplitude decreased for easier questions in which GARs were high (Fig 5). In the joint condition, the OFB outcome affected not only the score obtained by self but also that obtained by the partner. Therefore, regardless of the magnitude of the question’s GAR, the correct OFB would have beneficial consequences for partners, and they themselves would have positive emotional valence. Thus, a stronger RewP amplitude was evoked regardless of the GAR’s magnitude. In contrast, in the individual condition, the OFB did not affect the partner in terms of the social factor, and the final judgment depended only on OFB. Therefore, results suggest that when participants correctly answered a difficult question with a low GAR, they felt positive emotional valence because they had better knowledge of facts compared with the public, but when they correctly answered a question with a high GAR, the effect of this emotional valence weakened and the RewP amplitude became smaller. As mentioned above, RewP amplitude was strongly observed in correct OFB after incorrect PFB regardless of ExpCond. This result negates the fact that RewP was sensitive only to simple task-dependent rewards. These results suggest that RewP is sensitive to positive affective valence, such as feelings of superiority and satisfaction, as well as to simple task-dependent rewards.

### Loss- and gain-related P300

Our results showed that the loss-related P300 amplitude was larger in the individual condition than in the joint condition. Previous studies have not provided satisfactory consensus on the relationship between the modulation of loss- and gain-related P300s and personal responsibility. As mentioned in the previous subsection, earlier studies have reported that FRN was sensitive to personal responsibility, but several studies did not focus on modulation of the feedback P300s, possibly because the P300 is sensitive to various experimental and cognitive processes, and it was difficult to form a satisfactory hypothesis among experimental tasks and their reports. In fact, various studies, not just feedback-related studies, have indicated that P300 reflects multiple cognitive processes. Regardless of valence, feedback-related P300 is greater for unexpected outcome than for expected outcome [3, 11, 52, 53], indicating that P300 amplitude is sensitive to feedback probability. In addition, the typical P300 component is basically known to reflect attentional responses [51, 63–65], is associated with motivational/affective processes [11, 85, 86] and is modulated by interpersonal relationships [66]. Loehr et al. [26] reported that the P300 amplitude evoked by own errors (resulting from one’s own action) was larger than that evoked by joint errors (resulting from the coordinated actions of two people) in cooperative work. They claimed that allocation of attentional resources was greater in the individual setting in which they have full control compared with outcomes they share or cannot control. This result was consistent with the findings of the present study, in which errors in the individual condition were larger than those in the joint condition, suggesting that more attentional resources were allocated to individual errors. In addition, larger gain-related P300 was observed in one’s correct OFB after incorrect PFB than in the correct PFB. This result also suggested that P300 amplitude was modulated by increased allocation of attentional resources. When participants observed the preceding incorrect PFB, they recognized that the question was a difficult one that their partners could not answer correctly. Therefore, allocation of attentional resources for their OFB after the incorrect PFB was larger than those after the correct PFB may result in increased P300 amplitude.

Statistical analysis revealed that gain-related P300 correlated significantly negatively with the SCR (Table 7 and Fig 6C). Moreover, negative correlation between gain-related P300 and SCR was considerably larger in correct OFB after correct PFB than in incorrect PFB. Gain-related P300 amplitude after correct PFB was attenuated more strongly with increasing SCR than that after incorrect PFB (Fig 6D), i.e., the degree of attenuation of gain-related P300 after incorrect PFB was small. P300 is generally considered to reflect the magnitude of the attentional allocation to the stimulus [51, 63–65]. Therefore, the result suggested that the magnitude of the attentional allocation to OFB was modulated by the magnitude of SCR in the corresponding question. The task in the present study was a four-choice question. Therefore, if participants had the knowledge to answer the question correctly, they did not allocate attentional resources to OFB because they were strongly convinced of the correct OFB outcome. In contrast, if participants did not know and were not confident about the correct answer to the question, they would allocate more attentional resources to the OFB. However, attenuation of attentional allocation weakened on OFB after incorrect PFB. By observing incorrect PFBs, participants reaffirmed that the question was more difficult than they had originally assumed. As a result, allocation of attentional resources did not decrease more than correct PFB even if the SCR for the question was high.

The larger gain-related P300 after incorrect PFB appeared to be increased allocation of attentional resources because observation of the incorrect PFB led to recognition that the question was more difficult than they had originally assumed. Larger loss-related P300 in the individual condition can be explained by increased allocation of attentional resources to the OFB under the condition in which participants fully controlled the outcome. The negative correlation between correct OFB and SCR can be explained by the decrease in allocation of attentional resources to OFB depending on the increase in SCR. It appears that P300 was sensitive to conditions in which the participant might receive some part of negative emotional feeling (e.g., anxiety [87, 88]). However, note that modulation of the P300 amplitude differs from that of the FRN in its basic mechanism. A stronger FRN was elicited by negative feedback in a condition where participants felt socially responsible, but P300 modulation was observed not only in the incorrect OFB but also in the correct OFB. The P300 modulation itself did not reflect negative affective valence; it rather suggests reflection of the allocation of attentional resources caused by the subjective task difficulty of the negative outcome.

## Conclusions

In the present study, we comprehensively analyzed the effects of subjective/objective task difficulties (i.e., SCR and GAR) and social factors (i.e., joint/individual conditions and correct/incorrect PFB) on feedback-related potentials. Although many previous studies have reported that FRN/RewP and P300 are sensitive to feedback probability, we separated feedback task difficulties into subjective and objective task difficulties and focused on the variation of each task difficulty and feedback-related ERPs. The study showed that subjective task difficulty sensitively modulated the amplitude of gain-related P300, suggesting that it was sensitive to modulation in allocation of attentional resources to OFB. Objective task difficulty sensitively modulated the amplitude of RewP after incorrect PFB. RewP was more sensitive to positive affective valence, such as feelings of superiority over the partner, than to task-dependent rewards that participants themselves received. In addition, we found a more significant correlation between RewP amplitude and objective task difficulty in the individual condition than in the joint condition, suggesting that the RewP amplitude changes depending on one’s superiority compared with the public. In contrast, FRN was more negative in the joint condition than in the individual condition, suggesting sensitivity to the sense of social responsibility participants felt toward their partners. These are worthwhile research results of a comprehensive study of feedback-related brain potentials and subjective/objective task difficulties and social factors.

## Supporting information

S1 Appendix Experimental scripts. (ZIP)

S2 Appendix Statistical script. (R)

S3 Appendix Experimental data. (XLSX)
